# The challenges of donor engagement with faith-based organizations in Cameroon’s health sector: a qualitative study

**DOI:** 10.1093/heapol/czab006

**Published:** 2021-02-14

**Authors:** Sibylle Herzig van Wees, Michael Jennings

**Affiliations:** UGHRIS—Uppsala Global Health Research on Implementation and Sustainability, Department of Women's and Children's Health, Uppsala University, Uppsala, Sweden; Department of Global Public Health, Karolinska Institute, Stockholm, Sweden; Department of Development Studies, SOAS University of London, UK

**Keywords:** Faith-based organizations, FBOs, health sector, donors, advocacy

## Abstract

Substantial global advocacy efforts have been made over the past decade to encourage partnerships and funding of faith-based organizations in international development programmes in efforts to improve social and health outcomes. Whilst there is a wealth of knowledge on religion and development, including its controversies, less attention has been payed to the role that donors might play. The aim of this study was to describe and analyse the engagement between donors and faith-based organizations in Cameroon’s health sector, following the implementation of the Cameroon Health Sector Partnership Strategy (2012). Forty-six in-depth interviews were conducted in selected regions in Cameroon. The findings show that global advocacy efforts to increase partnerships with faith-based organizations have created a space for increasing donor engagement of faith-based organizations following the implementation of the strategy. However, the policy was perceived as top down as it did not take into account some of the existing challenges. The policy arguably accentuated some of the existing tensions between the government and faith-based organizations, fed faith-controversies and complicated the health system landscape. Moreover, it provided donors with a framework for haphazard engagement with faith-based organizations. As such, putting the implications of donor engagement with FBOs on the research map acknowledges the limitations of efforts to collaborate with faith-based organizations and brings to the surface still-remaining blinkers and limited assumptions in donor definitions of faith-based organizations and in ways of collaborating with them.

**KEY MESSAGES**

- There has been a strong global narrative of the benefits of partnering with faith-based organizations to improve social and health outcomes. This narrative has led to a national policy in Cameroon—The Cameroon health sector partnership strategy (2007–2015)—with the aim to strengthen collaboration between government and partners, including the faith-based sector.
- The policy led to increased engagement of faith-based organizations in the health sector and donors made these key recipients of substantial amount of funding.
- However, this research shows that the policy did not take into account some of the realities in practice, including the tensions between the government and the faith-based sector. It also shows that donors did not fully adhere to the framework and instead haphazardly engaged with faith-based organizations often on the basis of pragmatism.
- Consequently, some of the partnerships arguably complicated the health system landscape, increased the tensions between faith-based organizations and the government and arguably contributed to faith controversies.

## Introduction

Western biomedical health has been a site of faith-based and religious-linked engagement for over a century in sub-Saharan Africa (and longer elsewhere), before even the onset of colonial regimes (Jennings, 2013a). Yet, there have been significant changes in the importance of religion and international development and more specifically the role of faith-based organizations’ (FBOs) in development. In fact, today, FBOs that operate in the health system in Sub-Saharan Africa have a seat at the policy table in many countries, including Cameroon. Some have argued, and donors often believe, that they have now ‘done faith’: recognized its role and advocated for the importance of FBOs in the health system. This view, we suggest, fails to acknowledge the limitations, still-remaining blinkers and limited assumptions in donor definitions of faith-based organizations and in ways of collaborating with them. Here, we seek to highlight some of these limitations, and the consequences of being so limited. In this article, we focus on Christian faith-based organizations that play a formal role in the delivery of health services, the training of health staff and operate as umbrella organizations that facilitate these activities in the health system in Cameroon. This sector and group of actors in Cameroon have been significantly invested in by international donors—multilateral, bilateral, private foundations and missionary networks—particularly over the past decade. We highlight in the discussion some of the challenges, the missed opportunities and the failures of this engagement, and how this has affected those health-focused faith-based organizations, which in turn impact on the ways in which donors support (or fail to adequately support) the strengthening of health systems and public health in Cameroon.

### Narrative of ‘FBOs’ in international and national policy discourse

Donors turned their attention to the role of faith-based organizations from the late 1990s, a gaze that acquired greater urgency following the 9/11 attacks in 2001 and with it impetus to donors that engagement with religious and faith-organizations and leaders was an essential foreign policy tool in fragile and weak states (Clarke *et al.*, 2007). organizations like the World Bank, the British development organization DFID and others sought to understand what characterized the FBO, which types of organizations they could work more closely with, and the challenges that might arise from engagement with religion in development. Underpinning this new interest, divisive in donors as it was, was an understanding that faith actors could contribute in distinctive ways, and reach communities in ways that their secular counterparts could not (Aiken, 2010; Ware *et al.*, 2016; James, 2011).

The former president of the World Bank, Jim Yong Kim, took a clear stance on the importance of religion in their programmes and policies and the value of the moral imperative:

Faith leaders and the World Bank Group share a common goal – to realise a world free of extreme poverty… The moral imperative can help drive the movement to end poverty by 2030 … These commitments from religious leaders come at just the right time – **their actions can help hundreds of millions of people lift themselves out of poverty** [our emphasis]. (World Bank 2015)

Various UN institutions have followed a similar narrative and have actively sought to engage with religion in their development work. In the early 2000s, UNFPA, for example, sought to collaborate with faith leaders in their activities to improve global reproductive health. In 2014, they held a roundtable consultation titled ‘Religion and Development Post-2015: Challenges, Opportunities and Policy Guidance’ (UNFPA, 2014). Although they do discuss some controversies, especially with regard to family planning and religion, UNFPA advocates for a partnership with religious institutions (UNFPA, 2014).

At a time where basic needs are becoming increasingly harder to provide for more than half of the world’s population, we can no longer avoid acknowledging the parallel faith‐based development universe, **which reaches so many and provides so much** [our emphasis]. (UNFPA, 2014: 3)

The arguments in favour of stronger engagement with the faith sector in part reflected an acknowledgement of the growing evidence around the scale of its activities (Olivier and Wodon, 2012b; Kagawa *et al.*, 2012). Some data have suggested the faith sector provides around 50% of education and health services in sub-Saharan Africa (James, 2011), despite debate surrounding the source of the data (Olivier and Wodon, 2012a). Faith-based organizations played a huge (albeit also problematic) role in the response to HIV, for example, providing care (at a time when treatment was unavailable) as well as supporting people living with and affected by the virus, and promoting behavioural change (Garner, 2000; Green *et al.*, 2002). Indeed, the Catholic Church alone was estimated to be running around a quarter of global HIV and AIDS services in the mid-2000s (UNAIDS, 2012). But the pro-engagement arguments also considered the distinctive contributions faith-based and linked organizations could make, as a result of their faith identity. Faith-run institutions and services are more likely to be embedded in communities and better able to reach the poor. Faith institutions could create stronger and deeper social capital, established on and reinforcing trusting relationships between service providers and communities. Such organizations straddle the hyper local (embedded, physically in many cases, within communities in ways that few other institutions, even the state, could lay claim to), and at the global (through membership of globalized networks of faith). Sharing a world view as well as a shared (literal and metaphorical) language of development, health and wellbeing, and possessing temporal as well as spiritual power, they can create a particular form of ‘sticky trust’ (Jennings, 2013b), as well as act as powerful agents of social change (James, 2011; Jennings, 2013; Ware *et al.*, 2016). Moreover, religious leaders as a broker in communities were seen as invaluable (Narayan *et al.*, 2000), an active role of religion in society highlighted by Ellis and ter Haar (2004). Backed by such arguments, the international development sector largely bought into the idea of FBOs as a ‘powerful but flammable fuel for change’ (James, 2011: 110).

However, with growing advocacy for collaboration with FBOs, there was an emergence of research that was commissioned by donors to better understand how the sector was constituted and how faith and religion made specific contributions. Multilateral and bilateral donors engaged with religion and development in different ways, ranging from the creation of knowledge platforms (Switzerland^1^, Netherlands^2^, Sweden^3^, World Bank and Germany^4^) to the development of a 3.5 million 5-year research programme on Religion and Development. Various publications, including the World Bank’s two Volume publication on Strengthening faith-inspired health engagement (Olivier and Wodon, 2012b,c), as well as the contribution of religious entities to health in sub-Saharan Africa study (Schmid *et al.*, 2008) commissioned by B & M Gates Foundation were produced.

The knowledge exchange platforms, the commissioned research by donors and peer-reviewed literature that emerged during that time has engaged with important questions surrounding religion and development and has challenged the simplification of the actors and the assumptions about distinctive features (Lipsky, 2011; Leurs, 2012; Ware *et al.*, 2016). Also, there has been a growing acknowledgement of the complex relationship between donors and FBOs and a historical exploration of some of those relationships (Braley, 2014; Marshall, 2015). Concerns were raised about the increasing allocation of funding to FBOs despite weak evidence or guidance to support such engagement (Olivier and Wodon, 2014). The quality of evidence and types of evidence produced has further been questioned (Olivier, 2016).

Similarly rarely interrogated by donors are the ways in which faith and religious teaching can shape action and approaches. Through the use of contracts that emphasize which specific parts of work are being supported, through expectations on organizations that they will subscribe to agreed standards and services, donors take confidence that they have been able to construct strong, impermeable firewalls between the ‘faith’ and the ‘medical’ sides of FBO activity. Yet as the work of Hovland (2008) shows so clearly, these firewalls are imaginary, not the fixed and impermeable constructions of contract law and rigid separation of functions that is supposed. Where medical professionals have been called to their role through religious conviction, how can one draw a clear boundary between the spiritual and the secular? This view has also served to limit thinking about the ways in which faith and spirituality work to a set of overtly faith-based actors, further restricting acknowledgement of the role played in social and political life as a whole. Across much of sub-Saharan Africa, faith and spiritual beliefs form an absolutely central part to the ways in which people think, act and make decisions (Ter Haar and Ellis, 2004). Decisions made by health ministers, by physicians, by others working in the health sector, are drawn from their spiritual understandings and beliefs as much as any medical training or government experience—but where is that accounted for in donor (and, indeed, in many academic) understandings of policy and practice? Too often donors and analysts fall into the trap Sen (2006) does, when social action undertaken by people of faith is ascribed to political and social initiatives, quite separate from faith, as if people, as well as institutions, can set up clear firewalls between their own beliefs and public action.

Donors have sought to remove their faith-blinkers, and have managed to do so in some ways. But donor vision is still limited: focused on a narrow range of organizations; underscored by assumptions as to clear divisions in separation of faith and development action; and haphazard in the way it engages with FBOs. At the same time, they have tried to set up processes which are ‘faith-blind’, confident in their firewalls and contracts’ ability to constrain the impact of faith on action, thereby simultaneously seeking to open their eyes to faith and narrowing their gaze to exclude much from that vision. The consequence has been the prevalence of an optimistic narrative, which sees faith-based health actors as critical to national health service provision, even as it smooths out potential problematic areas and seeks to diminish the actual role of faith and religious thought in those organizations. Hershey (2016) has pointed to the way in which FBOs themselves write out faith in the narrative in order to appease secular-instincts of donors, but as this paper shows, donors too have been overly confident in their assumptions that religion and social service action can be easily divided into separate spheres by those organizations.

### Cameroon health sector partnership strategy

Cameroon was selected as an example to explore the relationship between donors, the government and FBOs because of limited literature on FBOs in Cameroon and because it makes an interesting case study due to its specific strategy that created a framework to increase donors’ engagement of FBOs. Global advocacy efforts culminated in the development of the Cameroon Health Sector Partnership Strategy (MOH, 2012). The Strategy was funded by the European Union, the French and German bilateral agencies, UNICEF and WHO. The strategy paper specifically recognizes the importance of private and non-state actors or faith-based in the health domain. The policy document is the result of a consensus reached by the steering committee. It includes a collaboration framework with non-state actors, including FBOs in the health system. It also highlights the importance of contracting of FBOs and refers to a legally binding contractual document that sets the framework for the collaborations between the government FBOs. The strategy encourages permanent development and a sustainable financing approach for FBOs, this includes putting in place a process to improve the financial situation, defining the rules and procedures of granting subsidies and instituting plans to develop and manage health structures. There were five aims: (1) put in place the legal institutional framework and technical support; (2) implement the contracting approach between public and FBO sector; (3) putting in place a new framework of relations among stakeholders; (4) supporting monitoring and evaluation; and (5) assessing the partnership and contracting approach. The strategy served as a framework for donor engagement of FBOs in selected large-scale programmes, including the French debt relief programme that invested millions of Euros into FBOs. The strategy was officially evaluated by an external consultancy group in 2016. However, the results of the evaluation have not been made publicly available. This research aimed to explore the perceptions and experiences of donors, FBOs and the Ministry of health of the Cameroon Partnership Strategy and how it affected their activities in the health sector^5^.

## Methods

### Study design and setting

This study took place in Yaoundé, the North-West and South-West and the East regions of Cameroon. Despite the country’s vast natural-resource wealth and a relatively robust economic performance over the past two decades, the health outcomes of the Cameroonian population have yielded disappointing results. The under-5 child mortality has decreased slightly,^6^ yet improvements lag behind global performance, as well as similar economies in sub-Saharan Africa (Singh *et al.*, 2013; DHS, 2019). The under-5 mortality rate lies at 84 deaths per 1000 children (UNDP, 2020), and maternal mortality, at 596 per 100 000 live births, is amongst the highest in the world (UNDP 2019). Life expectancy has remained stagnant at 54 years from 1990 to 2013, only marginally higher than life expectancy in conflict-affected countries such as the Central African Republic. Health outcomes are the worst in rural areas throughout the country (DHS, 2019). Cameroon’s political economic environment has been plagued by poor governance and a strongly centralized state (DAHLIN, 2019). In a context weighed down by some of the worst health indicators in the world, as well as a political climate of an authoritarian state with a record of abuse of public funds, non-state actors such as FBOs have thus caught the attention of donors investing in Cameroon. Over the past decade, FBOs throughout Cameroon have been recipients of various forms of aid, ranging from large-scale programmes—such as the World Bank’s $20 million per year project Performance-Based Financing (PBF) initiative (over a period of several years); multilateral aid by the UNFPA; or programmes from bilateral actors such as the French and German Cooperation and the United States—to receiving large sums from non-governmental organizations (NGOs) and international missionary networks. Most FBOs in Cameroon are officially part of the health system through memorandums of understanding with the Ministry of Health.

### Sampling

In-depth interviews were conducted with officials of the Ministry of Health (*n* = 9), FBOs (health providers, health professions schools and networks, staff working at faith-based health organizations) (*n* = 22), and donors in Cameroon and international headquarters (*n* = 15). Interview respondents in the donor category were affiliated with the Deutsche Gesellschaft fuer Internationale Zusammenarbeit (GIZ), the French bilateral cooperation, the World Bank, UNFPA, UNICEF, World Health Organization, The Global Fund, Elizabeth Glaser Pediatric Aids Foundation and The President's Emergency Plan For AIDS Relief (PEPFAR). The departments of the respondents from the Ministry of Health cannot be named because they could otherwise be identified and anonymity was guaranteed. Faith-based organizations were affiliated with three faith-based networks: the Catholic, the Protestant and Fondation ad Lucem (FALC). Respondents were affiliated with Catholic health providers, The Baptist Convention Network, Presbyterian health providers, and Evangelical-Lutheran health providers. Any study of faith-based organizations in Cameroon touches on some sensitive issues including a well-known corruption scandal that involved both the government and selected faith-based organizations. Moreover, the relationship between faith-based organizations and the state is complex as this study will show. Because of this sensitivity and given the authoritarian nature of the Cameroonian state, a detailed list of organizations and their locations are purposively omitted to protect the anonymity and ensure confidentiality of the research participants. Most donors wanted to remain anonymous. Purposive sampling of Ministry of Health staff and donors was applied to recruit actors that work with FBOs. Convenience sampling and snowball sampling was applied to recruit faith-based organizations and staff working in selected FBOs in three regions in Cameroon.

### Data collection

This research draws on 46 qualitative in-depth interviews. Data were collected between 2015 and 2017. Data was collected in Yaoundé and the North-West and South-West region and the East. Data collection was interrupted do to conflict in those areas. Eleven interviews were conducted via video Skype due to the conflict in the region. The regions were selected because there are many FBOs operating in the health sector in that region, which are funded by international donors. There was also an element of convenience sampling involved, these were sites with whom the researcher was familiar due to previous employment in the region. Interviews were conducted in English but participants had the option to either conduct the interview in French or English. The thematic guide centred around the exploration of examples of collaboration between donors and FBOs. Questions included, perceptions on the strategy, why, how and with what implications collaborations between donors and FBOs took place.

### Data analysis

Interviews were transcribed verbatism and transcripts were shared with the interviewee for quality control. Transcripts were imported into Nvivo11 and analysed with the assistance of the software. Three rounds of coding (Saldaña 2015) were conducted by two researchers to increase the quality of the analysis. The coding processes culminated in hundreds of codes and a total of nine categories. The coding tree (see Table 1)—i.e. the creation of categories and the agreement of themes—was agreed upon in the research team. The write up of the results section follows the structure of the coding tree.

**Table 1:** Categories and themes following process of qualitative coding.

Accentuating tensions: ‘They treat us like a business but we are doing the job of the government’	FBOs perceive themselves as unvalued partners in the health system
	FBOs have no legal framework
	FBO-Government contracts are not respected
	FBOs a silent and invisible partner
Donors’ haphazard engagement of FBOs	FBOs are a good alternative to the state
	FBOs constitute the health system
	Faith-blind engagement
	FBOs are well-funded health providers—easy to partner with

### Ethics

Informed written consent was obtained from all participants. The study received ethical approval from the Cameroonian Research Committee (No. 2015/08/638/CE/CNERSH/SP). For ethical reasons and fear of personal and political consequences, two Cameroonian data collectors and those assisting with this research choose not to be part of the write up of this paper although this piece of work would have not been possible without them. Moreover, we have omitted details of the participants such as name of organization, position and gender to protect their anonymity.

### Reflexivity

There are some implications to conducting research as an outsider or with a so-called foreign gaze. In some instances, it could be argued that an outsider cannot fully capture the complexity of a local reality. Moreover, the data one collects is likely to be influenced by the history (in this case of colonialism) and gender imbalances (for example it may be challenging to get a senior official to talk to a young white researcher). While the background of the researcher has arguably influenced the data collection, certain factors mitigated these limitations. SHvW was an employee of the German bilateral cooperation from 2009 to 2012 in Yaoundé, Cameroon. SHvW has developed a solid network of researchers who were involved in this study. Moreover, years of experience in the health sector allowed a foreign gaze while also having a good understanding of the contextual specificities. Access to in-depth findings from donors—many of whom SHvW knew to some extent for years—led to the fact that they were open to share their challenges and thoughts. The authors of this paper see, and approach, faith institutions as institutions of power, wielding authority (temporal as well as spiritual), making claims as to legitimacy, and having access to and control over resources, which can be problematic as well as offering opportunities, just as any other institution of power does. The authors do not hold religious positions in relation to any particular faith teachings on issues such as reproductive and sexual health, rights around sexuality and other related areas. We have approached faith institutions and faith-based health providers neither as adherents nor critics of religion, but subject to the same dilemmas of conducting objective research that all researchers face whether engaging with institutions that reflect religious-, political- or other values.

## Findings

### Accentuating tensions: ‘they treat us like a business but we are doing the job of the government’

The analysis of the interview data revealed a contrary reality to the narrative promoted by multi-lateral and bilateral donors and the national policy makers. Faith-based organizations interviewed for the purpose of this research did not perceive themselves as valued partners in the health system. Despite the Cameroon Health Sector Partnership Strategy (2007–2015) there are legal barriers that hinder an effective partnership. For example, one major challenge that hampers an effective relationship between FBOs in Cameroon and the state is the fact that they have to pay the same tax as a private business, although they provide services to the Cameroonian population that the state should be able provide. In fact, all non-state actors in Cameroon fall into this bracket (Interviews #1/2/4/5/6 FBOs). In the words of an FBO: ‘They treat us like a business but we are doing the job of the government’. (Interview#5 FBO) Interviews with the Ministry of Health confirmed this problem is indeed a challenge that needs to be addressed. ‘We continue to ask them to pay tax. Some pay it, some don’t manage. It’s a problem. We need to think about how to manage it’ (Interview#12 MoH). A further critique from FBOs is that despite the legal contractual framework, contracts are not actually respected. In the words of an FBO: ‘They [the Ministry of Health] are supposed to pay us a visit. They are supposed to pay us CD2 money [French debt relief funding]. But have we seen them. No, they don’t come.’ [Interview #4 FBO].

In terms of participation in policy processes, both FBOs and donors express concern. Faith-based organizations question the actual voice they have, although they may be invited to the policy table and to specific health planning events they doubt their influence. Several FBOs claim that although they were invited to a midwifery curriculum planning event, they did not actually have a say:

Yes, they [Ministry of Health] invite us sometimes. For planning maybe. We go, we say what we think but they don’t actually listen. [laughs]. We made curriculum suggestions for the midwives. They accepted the changes at the workshop. But when they printed the curriculum they did as they wanted. So participate yes but do we have a voice? No. [laughs]. [Interview#11 FBO]

A further factor that illustrates that FBOs are not as endorsed as the international policy discourse and national policy strategy suggests, is reflected in donor’s concerns about FBOs actual independence. A donor voiced concerns about the FBOs’ role in the steering committee [that includes FBOs and the Ministry of Health] for a programme for which FBOs were identified as the main recipient:

All we receive are requests or communication from the Ministry of Health; it’s like the faith-based networks [FBOs] are not even part of the steering committee. We can’t talk to them. [Interview#22 Donor]

Another donor claims that since the time they have spent in Cameroon, which is two years, they have always seen the FBOs in the meetings but no one has ever actually said anything [Interview#21 Donor].

A last topic that emerged from the data, suggests that FBOs in rural areas are not actually represented by their networks, neither do they know what is going on at national policy level. Thus reiterating the idea of them feeling invisible at national level. In the words of an FBO:

They [the protestant faith-based network CEPCA^7^] should represent us but we hear very little from them. [Laughs] We don’t know what they do in Yaoundé [Laughs]. [Interview#8 FBO]

Faith-based organizations at the district do not feel that their concerns are being heard, nor do they feel included in the decision-making processes of CEPCA. For example, several CEPCA members feel that they have not been involved at all in decision-making concerning the use of funding [referred to as CD2] that was earmarked for the benefit of all FBOs in the country. One interviewee suggests that CD2 benefitted only those at the central level, with some very limited benefits to the faith-based health providers in the periphery [Interview#35 FBO]. Questions about CD2 were generally met either with cynicism or with distain. An FBO explains,

We provide care for a large part of the population, we are a district hospital, in fact we are one of the biggest FBOs in the country (!) but we have not ever been asked about how we think the CD2 [French debt relief funding specifically allocated to FBOs only] money should be used…it’s like we are invisible. [Interview#41 FBO]

This and further qualitative interview data consistently suggest very poor communication between faith-based networks at the central level, faith-based network health coordinators (at regional level), and the faith-based providers. The data presented in this section suggest that despite a policy framework that endorses the role of FBOs in the provision of health care in the country, these actors do not consider themselves as part of the national health system. They are treated like a business by the state, their opinions and views are often ignored and there are questions to their level of participation even at the central Ministerial level. In other words, many FBOs consider themselves as invisible actors.

### Donors’ haphazard engagement of FBOs

International donors endorse collaborations with FBOs and have actively supported the development of the Health Sector Partnership Strategy (2007–2015), which encourages FBOs’ inclusion into the health system (MOH, 2012). However, the findings in this research show that even donors’ reasons for engaging FBOs in their health programmes varied from the international narrative of these organizations whereby FBOs are portrayed as an added benefit to health programmes. In fact, donors engage FBOs for a variety of reasons and often this is haphazardly rather than informed by an international strategy.

Donors repeatedly noted that investing in FBOs is a good alternative to the state. For example, in the French debt relief programme—which initially only provided funds to the state—the French government needed an alternative actor to receive this funding due to a history of poor governance of those funds [Interview#29 donor]. The French cooperation was under pressure to spend debt relief funds. Similarly, donors selected to collaborate with FBOs in rural areas for the same reason, to bypass the state and with that reduce the risk of poor governance of funds [Interview#27 donor].

We [donor] spent years trying to negotiate with the government what the money would be spend for. When we invested money… [hesitates] a lot of it disappeared. We still don’t know where it is [laughs]. So, the faith-based networks were a good alternative. It’s like they were the solution to our problem… [Interview#29 donor].

To add to the governance argument, data analysis revealed that donors like FBOs because it is easy to get results and invest in something long term:

Working with faith-based providers is sometimes easier than working with the government. You can get results. Plan something a bit long term. And… there is more trust. Less money disappears [laughs] [Interview#31 donor].

A second reason for engagement with FBOs at the district level was because donors claim that they constitute the health system. In other words, they are not partners because of any special attributes but rather because there is no public counterpart [Interviews #23 and #17 donors].

We support district medical teams, and it is up to them to select whoever they think is most suitable to participate in our programmes. Faith-based organizations happen to be part of the district system. [Interview#27 donor]

In treating FBOs the same as the public counterparts, it appears that donors take a ‘faith-blind’ approach. The following extract further confirms this finding:

In our programme everyone is treated as equal […] ***if they choose not to do family planning or don’t offer vaccination service, that’s fine***, we don’t restrict them from the programme. We have others where they offer the entire pack of services but not modern contraceptives and that’s fine. [Emphasis ours, Interview#24 donor]

In other words, the donor accepts the exclusion of family planning, which in turn could have implications on access to those services for women. In a similar vein,

We have some data on this [the differences of performance between public and FBO providers] but ***we haven’t had the pressure or interest to analyse the data we have***. What we are interested in is the general development. [Emphasis ours, Interview#42 donor]

An unpublished report by an anonymous donor showed clear differences between faith-based providers providing family planning services and public providers. In the words of that donor:

It would probably be good to think about those differences more. We haven’t had the time consider it. [Interview#29 donor]

The extracts show that some donors engage with FBOs without much consideration of their faith attributes, even though some of these programmes engage with well-known faith-controversies in reproductive health and religion.

A third reason for donors funding of FBOs is because some of them are well-established health providers. Hence, because they have a long history of having received funding from various donors, they have a good technical level in some areas such as HIV/AIDS and reproductive health. Therefore, they make easier partners because health programmes do not have to begin with the basics [Interview#22 donor]. A number of donors as well as missionary networks such as the St. Francis Sisters and the Baptist Convention, exclusively collaborate with particular FBOs. The Baptist network regularly turns down funding opportunities because they receive so much funding and cannot absorb it all; for example, they have turned down participation in the Global Fund although they were explicitly invited [Interview#30 donor; Interview#X FBO].

Whilst religion is presented as something that suggests a comparative advantage in international policy documents, this is not the case from interview data collected for the purpose of this research. One donor reasoned, ‘Yes, we collaborate with FBOs. But we don't consciously collaborate with them because of they are faith-based’ [Interview#27 donor]. Thus, it appears that is emphasized by the donors in question in international policy document about the added value of religion and its benefits for development, matters very little when programmes are implemented in practice.

## Discussion

Global advocacy efforts for increased partnerships with FBOs led to the development of the Cameroon Health Sector Strategy, which put at the centre the important role of FBOs in Cameroon’s health system. This provided donors with a framework to collaborate with FBOs in a wide range of health programmes, culminating in increasing engagement of FBOs in Cameroon. The aim of this research was to explore the perceptions and experiences of donors, FBOs and the Ministry of health on how the Cameroon Partnership Strategy impacted their development practice in the health sector. This study is qualitative and did not aim to evaluate the strategy but instead present views on its perspectives. The findings presented in this research show that the Cameroon Health Sector Strategy did not take into account existing challenges (e.g. existing legal barriers) at its conception. This may have accentuated some of the existing problems in that FBOs do not perceive themselves as an integral part of the health system, especially due to legal limitations. The results from this study show that FBOs feel invisible and underappreciated by the government. This echoes findings from a policy report that described the relationship between an FBO in Northern Cameroon and the government (Boulenger and Criel, 2012). The process of contractualization was very slow, the contracts were not respected, and the hospital’s agency was very limited (ibid.). The policy report also highlighted that the Health Partnership Strategy ignored previous arrangements between the government and the FBO but instead imposed a new and top-down strategy that did not build on the complex history of the relationship between these actors (Boulangier and Criel, 2012). The Health Partnership Strategy was evaluated by external consultants in 2016, the publication of those results have been impeded by the government. This is arguably because the results showed that the strategy was unsuccessful, as this research indicates. Arguably, the problem was the top-down nature of the policy, which was largely driven by external donors.

Furthermore, the strategy perhaps benefitted donors mostly by creating a framework for haphazard engagement of FBOs. Faith-based organizations were thus not engaged because of faith attributes but because they were a good alternative to the state. There are a complex array of implications of this. For example, there are examples whereby donors strengthen them and set them apart from the health system as described here and elsewhere (Green *et al.*, 2002, Herzig van Wees, 2020). This can be problematic because it creates a parallel system. This echoes Hearn’s (2002) work that showed that FBOs engagement by USAID in Kenya systematically undermined the role of the state. However, investing in FBOs could also be a good thing in that specific health initiatives are successfully and rapidly rolled out. Yet, donors may overestimate FBOs capacities and invest significant funding in them, which may have negative implications for FBOs. For example, selected Cameroonian FBOs who received large sums of debt relief funding have a very poor reputation today due to claims that they have abused funds (Herzig van Wees, 2019). However, instead of blaming FBOs for corruption, donor’s poor choice of engaging these actors based on assumptions should instead be discussed. A further implication of haphazard donor engagement of FBOs is when donors take a faith-blind approach as described above can affect the activities of FBOs. The above data shows that several donors were not concerned about faith-controversies but instead were more concerned about achieving results. This finding builds on research on religion and global health and faith-controversies in sexual and reproductive health (Tomalin, 2011; Tomkins *et al.*, 2015).

Literature on religion and family planning shows that the relationship between religion and family planning is complex. There are several debates at the interface between gender, faith and health care, and they regard family planning, child protection, violence against women, sexual and reproductive health and HIV (Tomkins *et al.*, 2015). It is well-established that religious beliefs can strongly influence individuals’ or a society’s attitude towards family planning, which in turn affects how decisions are made (Burket, 2006). Moreover, some Christian denominations, such as the Catholic faith, strongly oppose some contraceptive methods, such as the condom and other methods and only promote natural family planning (Tomkins *et al.*, 2015). There are also debates around engagement of religious actors in the fight against HIV/AIDS have received significant attention in the literature (Morgan *et al.*, 2013; Tomkins *et al.*, 2015). Resistance to implementation of family planning programmes in FBOs has been well-documented, and some have argued that this collaboration may take time and include compromises and challenging dialogues between donors and religious leaders (Barot, 2013; Aylward and Friedman, 2014; Duff and Buckingham, 2015). Thus, donor’s ‘faith-blind’ approach when collaborating with FBOs in sexual and reproductive health programmes and as described in this study is problematic. It can hinder women’s access to comprehensive services, and as the emerging literature on FBOs providing family planning suggests, if the controversial topic is dealt with in a constructive dialogue, FBOs can be an important vehicle to help improve access to all forms of contraception.

The consequence of donor instrumentalization, and limited understanding of faith engagement in development, and specifically health, is that they can all too easily miss, or misinterpret, what is going on. Donors may fail to hear the religious undercurrents framing discussions and decision-making, confident in their firewalls and in the surface presentation of ‘secular’ health-based language. Donors may fail to see the contradictions inherent in supporting some organizations at the same time as insisting on certain policies and best practice, because they miss the way the practice can be subverted or bent. In essence, by assuming there is a rigid separation between faith and ‘secular’ development, they fail to understand the complex and nuanced ways in which religion and faith can permeate all and shape outcomes. Donors have tried to take elements of faith action, faith teaching, and bend it to fit development and social service delivery, instrumentalising faith actors, leaders and teachings as if one can pick and choose which parts to take and which to leave. Engagement with faith organizations is rarely as simple as donors would wish.

At the core of it is the artificial divide between secular and faith. Attempts to classify organizations have largely failed because organizations are complex and fluid and because many FBOs are embedded in religious societies. Olivier notes at a mapping workshop in Uganda, a participant insisted that ‘all organizations in Uganda are faith-based’ (Olivier and Wodon, 2012a). In Olivier’s words ‘The Cartesian division between religious and secular has been imposed on development contests by the (northern) powers within development (and within the advocacy effort)’ (Olivier, 2016: 7). The evidence presented in this article highlights this issue, it shows that donors have tried to take elements of faith action, faith teaching, and bend it to fit development and social service delivery, instrumentalising faith actors, leaders and teachings as if one can pick and choose which parts to take and which to leave.

## Conclusion

Global advocacy efforts for increased partnerships with FBOs led to the development of the Cameroon Health Sector Strategy (2007–2015), which put at the centre the important role of FBOs in Cameroon’s health system. This provided donors with a framework to collaborate with FBOs in a wide range of health programmes. However, as this study shows the policy was perceived as failing to reflect the context and needs of the time. It also shows that donor’s haphazard engagement of FBOs is not much about a clear strategy but rather about pragmatic solutions in a challenging context. Exploring the influence of advocacy and donor driven policies is important because as discussed, there are implications that may undermine development progress. Instead, if we work off the premise that global advocacy efforts for collaborations with FBOs has an impact on practice, we gain a new perspective on how we should think about some of the controversies that the literature has raised about FBOs. Therefore, the more pertinent question regards how FBOs are shaped by the development process—in other words, how donors have engaged them and potentially *contributed* to some of the challenges with which the literature concerns itself.

## Footnotes

1 In 2002, the Swiss Agency for Development Co-operation (SDC) organised a conference with the title Religion and Spirituality: A Development Taboo?, resulting in several publications and followed by a series of workshops with NGOs. the documents are only available in German (the latest also in French) at www.eda.admin.ch/deza/de/home.html

2 The Knowledge Forum for Religion and Development Policy was established in 2005, to strengthen the policy dialogue between the Dutch Foreign Ministry and Dutch FBOs (Bartelink, 2016:113)

3 The first meeting on religion and development took place in 2009. More recently, SIDA took up the topic of religion in one of its Development Talks, a forum for knowledge and debate on global development cooperation. For more information on the seminar see: www.sida.se/Svenska/aktuellt-och-press/development-talks/dt-inbjuningar/religions-role-in-developmentcooperation/.

4 In 2016, the German Federal Ministry for Economic Cooperation and Development (BMZ) launched the International Partnership on Religion and Sustainable Development (PaRD)—an intergovernmental knowledge and coordination hub with the aim to ‘strengthen and institutionalize cooperation between governments, multilateral organizations, nongovernmental organisations (NGOs), academia, and religious actors’ (PARD, 2019). *International Partnership on Religion and Sustainable Development* [Online]. Available: http://www.partner-religion-development.org/about/partnership-principles/ (Accessed February 2019).

5 The aim was not to evaluate the effectiveness of the strategy but rather explore perceptions of it.

6 Approximately 16 more children out of every 1000 under the age of 5 in Cameroon (DHS, 2011). Enquête Démographique et de Santé et à Indicateurs Multiples (EDS-MICS)Yaounde.

7 CEPCA is the protestant network that represents all faith-based organizations at central level.
